# Tea Polyphenols EGCG and Theaflavin Inhibit the Activity of SARS-CoV-2 3CL-Protease *In Vitro*

**DOI:** 10.1155/2020/5630838

**Published:** 2020-09-16

**Authors:** Minsu Jang, Yea-In Park, Yeo-Eun Cha, Rackhyun Park, Sim Namkoong, Jin I. Lee, Junsoo Park

**Affiliations:** ^1^Division of Biological Science and Technology, Yonsei University, Wonju, Republic of Korea; ^2^Department of Biochemistry, Kangwon National University, Chuncheon, Republic of Korea

## Abstract

COVID-19, a global pandemic, has caused over 750,000 deaths worldwide as of August 2020. A vaccine or remedy for SARS-CoV-2, the virus responsible for COVID-19, is necessary to slow down the spread and lethality of COVID-19. However, there is currently no effective treatment available against SARS-CoV-2. In this report, we demonstrated that EGCG and theaflavin, the main active ingredients of green tea and black tea, respectively, are potentially effective to inhibit SARS-CoV-2 activity. Coronaviruses require the 3CL-protease for the cleavage of its polyprotein to make individual proteins functional. EGCG and theaflavin showed inhibitory activity against the SARS-CoV-2 3CL-protease in a dose-dependent manner, and the half inhibitory concentration (IC_50_) was 7.58 *μ*g/ml for EGCG and 8.44 *μ*g/ml for theaflavin. In addition, we did not observe any cytotoxicity for either EGCG or theaflavin at the concentrations tested up to 40 *μ*g/ml in HEK293T cells. These results suggest that upon further study, EGCG and theaflavin can be potentially useful to treat COVID-19.

## 1. Introduction

A new coronavirus disease (COVID-19) has become the most dangerous pandemic of this century causing over 20,000,000 reported infections and over 750,000 reported deaths as of August 2020. However, a vaccine is not yet available, and effective remedies to treat COVID-19 are still under development. Therefore, the infections and deaths due to COVID-19 will continuously increase until an effective vaccine or treatment is available. For this reason, various approaches to slow down the spread of COVID-19 need to be identified and developed.

Virus-specific enzymes are most often the main targets of antiviral medicines. For example, the thymidine kinase enzyme of herpesvirus is the main target of herpesvirus therapies, and thymidine kinase inhibitors including acyclovir and ganciclovir were developed to treat herpesvirus diseases [1]. Similarly, coronavirus-specific enzymes should be potential targets to treat coronavirus diseases. The 3CL-protease is regarded as the main drug target for coronavirus diseases [2]. Cleavage of viral polyproteins by proteases is a vital step in the life cycle of coronaviruses, and several viral proteases encoded in the coronavirus RNA are required for the maturation of the viral proteins [3]. The 3CL-protease is responsible for the cleavage of viral polyproteins of coronavirus and absolutely required for replication of the virus [4–6]. In light of its importance, the 3CL-protease is a target for antivirals, and several antiviral candidates were identified using a 3CL-protease assay [7–9]. Recently, chemicals targeting the SARS-CoV-2 3CL-protease have been reported to be effective to slow down the replication of coronavirus *in vivo* [10].

Green tea is a popular beverage, and many reports demonstrated that green tea has health benefits including cancer prevention [11, 12]. Epigallocatechin-3-gallate (EGCG) is the major ingredient in green tea and accounts for 50% to 80% of a brewed cup of green tea [11, 13]. EGCG or green tea showed a wide range of antiviral activity against adenovirus, influenza virus, zika virus, herpesvirus, and hepatitis virus [14–20]. In addition, a previous report demonstrated that green tea has antiviral activity toward coronaviruses [21]. Theaflavin is an active ingredient of black tea, a fermented or oxidized form of green tea [22]. Theaflavin also showed antiviral activity against influenza virus, herpesvirus, rotavirus, and coronavirus [23–25].

Since previous studies suggest that the active ingredients of green tea or black tea are effective to inhibit coronavirus 3CL-protease, we examined whether EGCG and theaflavin showed inhibitory effects on SARS-CoV-2 3CL-protease. In this study, we chemically synthesized the gene encoding SARS-CoV-2 3CL-protease using the published SARS-CoV-2 genome sequence and then utilized the protease assay to evaluate the inhibitory effect of EGCG and theaflavin.

## 2. Materials and Methods

### 2.1. Reagents

(−)-Epigallocatechin gallate (EGCG) (E4134, purity ≥ 95%) was purchased from Sigma-Aldrich (Saint Louis, MO). Theaflavin (No. 25129, purity ≥98%) was purchased from Cayman Chemical (Ann Arbor, MI). For preparation of EGCG auto-oxidation products (EAOPs), EGCG was dissolved in 200 mM PBS (pH 8.0) at a concentration of 5 mg/mL, and auto-oxidation of EGCG was carried out at 37°C for 0, 3, 6, 9, 12, and 24 hours according to a previous report [26].

### 2.2. Preparation of SARS-CoV-2 3CL-Protease

The nucleotide sequences for SARS-CoV-2 3CL-protease were obtained from the NCBI nucleotide database (http://www.ncbi.nlm.nih.gov, Ref Seq No : NC_045512.2), and the protein-coding sequence was chemically synthesized and cloned into a pBT7 vector by Bioneer (Daejon, South Korea). The plasmid encoding His-tagged 3CL-protease was transformed into BL21 (DE3) competent cells. His-tagged SARS-CoV-2 3CL-protease expression was induced by 0.1 mM IPTG at 18°C for 12 h, and soluble 3CL-protease proteins were purified with an Ni-NTA resin (Thermo-Fisher Scientific, Rockford, IL) according to the manufacturer's protocol. The purified 3CL-protease protein was dialyzed with dialysis buffer (137 mM NaCl, 2.7 mM KCl, 10 mM Na_2_HPO_4_, 1.8 mM KH_2_PO_4_, and 10% glycerol (pH 7.4)). The host strain BL21(DE3) (CP110) was purchased from Enzynomics (Daejeon, South Korea).

### 2.3. Protease Assay for SARS-CoV-2 3CL-Protease

A FRET-based protease assay was used to measure 3CL-protease activity [27]. Dabcyl-KTSAVLQSGFRKME-Edans was chemically synthesized (Anygen, Gwangju, South Korea) and used for the SARS-CoV-2 3CL-protease substrate. The 3CL-protease activity was performed at 37°C using 3CL-protein and FRET peptide in the reaction buffer (20 mM Tris-HCl (pH 7.5), 200 mM NaCl, 5 mM EDTA, 5 mM DTT, and 1% DMSO) for 5 h. For the inhibition assay, the purified 3CL-protease was incubated with EGCG or theaflavin for 1 h before the addition of substrate. The fluorescence was measured at 528 nm with excitation at 360 nm using a Synergy HTX multimode microplate reader (Biotek, Winooski, VT). Protease activity was calculated as the difference between the activity with 3CL-protease and the activity without 3CL-protease at the indicated time.

### 2.4. Cell Culture and Cytotoxicity Assay

HEK293T human embryonic kidney cells were maintained in DMEM (Welgene, Seoul, Korea) containing 10% fetal bovine serum (Thermo-Fisher Scientific, Waltham, MA, USA) and antibiotic-antimycotic solution (Welgene). Cell cytotoxicity was measured using the 3-(4,5-dimethylthiazol-2-yl)-2,5-diphenyltetrazolium bromide (MTT) assay. Briefly, equal number cells (2 × 10^5^ cells/well) were seeded in the wells of a 24-well plate and incubated in the presence or absence with EGCG or theaflavin. After 24 h incubation, MTT solution was added to a final concentration of 1 mg/mL, and the mixture was incubated for an additional 3 hours. MTT was purchased from USB Corporation (Cleveland, OH, USA). HEK293T (ATCC CRL-11268) cells were obtained from ATCC (Rockville, MD, USA).

### 2.5. Statistical Analysis

The results of 3CL-protease activity and MTT were evaluated by a 2-tailed Student's *t*-test using Excel software (Microsoft, Redmond, WA, USA). A *p* value of 0.05 was considered significant. For the calculation of half inhibitory concentration (IC_50_), the AAT Bioquest website program was used (https://www.aatbio.com/tools/ic50-calculator-v1). The coefficient of drug interaction (CDI) was calculated to determine the drug interaction between two different drugs. CDI is defined by the following formula: CDI = AB/(AxB) [28].

## 3. Results

### 3.1. Expression of SARS-CoV-2 3CL-Protease

**Figure 1:**
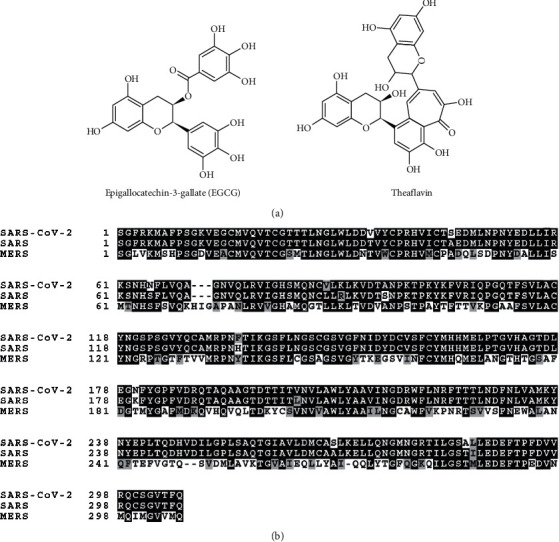
Amino acid sequence of SARS-CoV-2 3CL-protease. (a) Chemical structure of EGCG and theaflavin. (b) Amino acid sequence alignment of SARS-CoV-2 3CL-protease, SARS 3CL-protease, and MERS 3CL-protease.

**Figure 2:**
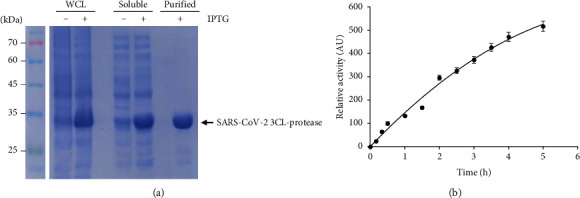
Expression of SARS-CoV-2 3CL-protease. (a) Purification of SARS-CoV-2 3CL-protease. Whole cell lysate (WCL), soluble fraction (soluble), and purified 3CL-protease protein (purified) in the presence or absence of IPTG were subject to SDS-PAGE. (b) Measurement of the protease activity of SARS-CoV-2 3CL-protease. AU, arbitrary unit.

Recently, the nucleotide sequence of the SARS-CoV-2 genome was published [29]. We aligned the SARS-CoV-2 3CL-protease peptide sequence with MERS 3CL-protease and SARS 3CL-protease (Figure 1(b)). Based on the amino acid sequence analysis, SARS-CoV-2 3CL-protease shares 96.08% identity with SARS 3CL-protease and 49.51% identity with MERS 3CL-protease (Figure 1(b)). Sequence alignment data indicate that SARS-CoV-2 3CL-protease is more homologous to SARS 3CL-protease than MERS 3CL-protease. We used the SARS-CoV-2 3CL-protease nucleotide sequence to synthesize the gene that encodes the 3CL-protease protein. In order to conduct the SARS-CoV-2 3CL-protease assay, we expressed a His-tagged 3CL-protease in bacteria by IPTG induction and purified the 3CL-protease by a His-tag affinity column (Figure 2(a)). SARS-CoV-2 3CL-protease is readily detected in the soluble fraction, and we purified the 3CL-protease for the 3CL-protease assay (Figure 2(a)). To identify potential inhibitors of 3CL-protease activity, we used the 3CL-protease assay. As expected, the 3CL-protease assay showed higher activity as the incubation time increased up to 5 h (Figure 2(b)).

### 3.2. The Inhibitory Effect of EGCG on SARS-CoV-2 3CL-Protease Activity

**Figure 3:**
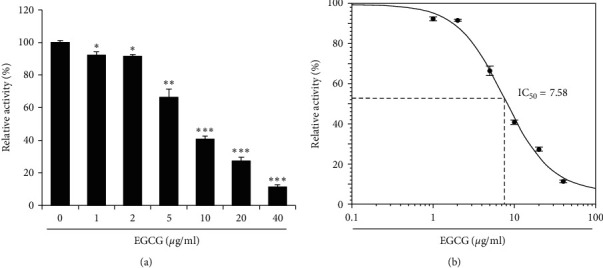
EGCG inhibits 3CL-protease *in vitro*. (a) Indicated concentrations of EGCG were incubated with 3CL-protease, and the 3CL-protease activity was determined. The 3CL-protease activity was performed in triplicate, and the mean and standard deviation are shown. (b) IC_50_ of EGCG was calculated and shown in the graph. Control vs EGCG treatment: ^*∗*^*p* < 0.05,^*∗∗*^*p* < 0.01,^*∗∗∗*^*p* < 0.001.

We examined the effect that EGCG has on the 3CL-protease activity by testing various concentrations of EGCG (0, 1, 2, 5, 10, 20, and 40 *μ*g/ml). We found that EGCG significantly inhibits 3CL-protease activity in a dose-dependent manner (Figure 3(a)). We calculated the half inhibitory concentration (IC_50_) of EGCG using the AAT Bioquest website program and calculated an IC50 for EGCG of 7.58 *μ*g/ml (Figure 3(b)). These results indicate that EGCG is an inhibitor of SARS-CoV-2 3CL-protease.

### 3.3. The Inhibitory Effect of Theaflavin on SARS-CoV-2 3CL-Protease Activity

**Figure 4:**
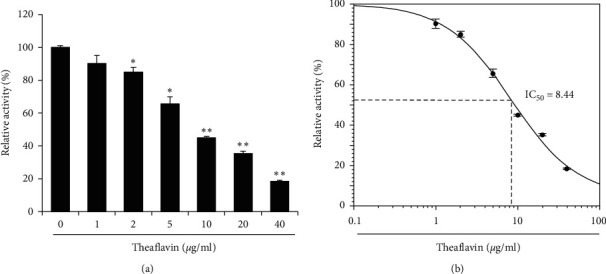
Theaflavin inhibits 3CL-protease *in vitro*. (a) Theaflavin was incubated with 3CL-protease, and the 3CL-protease activity was determined. (b) IC_50_ of theaflavin was calculated and shown in the graph. Control vs theaflavin treatment: ^*∗∗*^*p* < 0.01,^*∗∗∗*^*p* < 0.001.

Next, we examined the effect that theaflavin has on 3CL-protease activity by adding various concentrations of theaflavin (0, 1, 2, 5, 10, 20, and 40 *μ*g/ml) with 3CL-protease and performing the 3CL-protease assay. The results showed that theaflavin significantly inhibits 3CL-protease activity in a dose-dependent manner similar to EGCG (Figure 4(a)). The half inhibitory concentration (IC_50_) of theaflavin was 8.44 *μ*g/ml, slightly higher than that of EGCG (Figure 4(b)). These results indicate that EGCG is a more effective inhibitor of SARS-CoV-2 3CL-protease activity than theaflavin.

### 3.4. Additive effect of EGCG and Theaflavin

**Figure 5:**
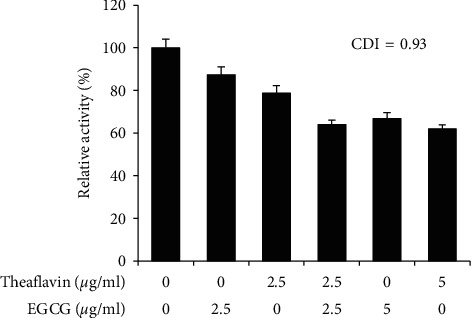
EGCG and theaflavin have an additive effect. Indicated concentration of EGCG and theaflavin were incubated with 3CL-protease, and the coefficient of drug interaction (CDI) was calculated.

Because EGCG and theaflavin both inhibit SARS-CoV-2 3CL protease, we examined whether EGCG and theaflavin together may have an additive or a synergistic inhibitory effect on 3CL-protease activity. We incubated EGCG alone, theaflavin alone, and EGCG/theaflavin and measured the inhibitory effect on 3CL-protease. Using the observed protease activity, we determined the coefficient of drug interaction (CDI) and calculated a CDI for EGCG and theaflavin of 0.93, indicating that EGCG and theaflavin had an additive rather than a synergistic effect (Figure 5).

### 3.5. Inhibitory Effect of EGCG Auto-Oxidation Products (EAOPs) on SARS-CoV-2 3CL Protease

**Figure 6:**
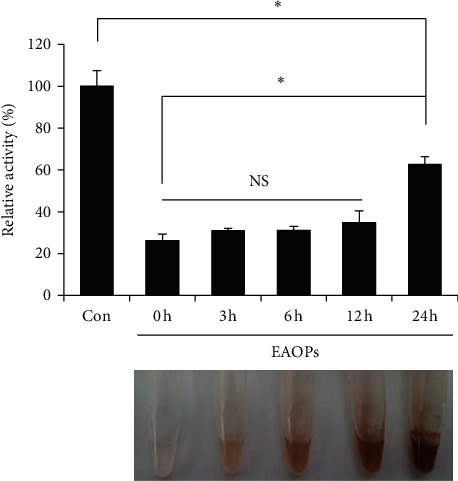
EGCG auto-oxidation products (EAOPs) show the inhibitory effect on SARS-CoV-2 3CL protease. Auto-oxidation of EGCG was carried out to produce EAOPs for the indicated hours. EAOPs (10 *μ*g/ml) were incubated with 3CL-protease, and the 3CL-protease activity was determined (top panel). “Con”, mock control. The mean and standard deviation are shown. ^*∗*^*p* < 0.05; NS, not significant. Time-dependent color changes of EAOPs were shown (bottom panel).

EGCG is susceptible to oxidation, and auto-oxidation of EGCG produces EGCG auto-oxidation products (EAOPs) in a time-dependent manner [26]. We examined whether EAOPs retain inhibitory activity on the 3CL protease. The color change indicates the production of EAOPs, and the inhibitory activity of EAOPs was not significantly changed up to 12 h (Figure 6). Although the inhibitory activity was decreased at 24 h, EAOPs still retain a significant inhibitory effect on SARS-CoV-2 3CL-protease (Figure 6).

### 3.6. Cytotoxicity of EGCG and Theaflavin

**Figure 7:**
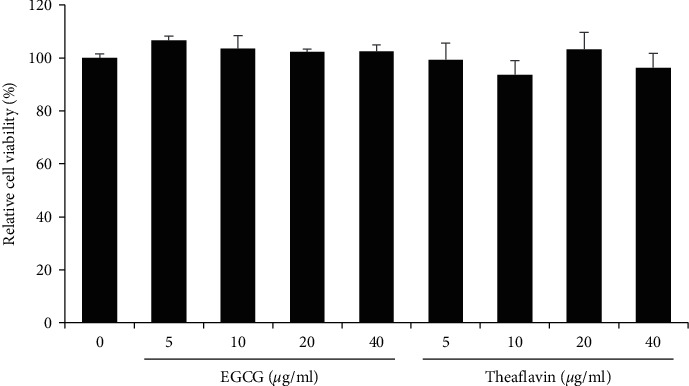
Cytotoxicity of EGCG and theaflavin. EGCG and theaflavin did not have significant cytotoxicity up to 40 *μ*g/ml. HEK293T cells were incubated with either EGCG or theaflavin for 24 h, and the MTT assay was performed to evaluate the cytotoxicity.

Previous reports showed that EGCG and theaflavin showed cytotoxicity on cultured cells [30, 31]. Therefore, we examined whether EGCG and theaflavin affected the cell viability of HEK293T cells. We incubated various concentrations of EGCG (0, 2.5, 5, 10, 20, and 40 *μ*g/ml) with HEK293T cells for 24 h and found that EGCG and theaflavin did not affect the cell viability significantly at any of the concentrations tested (Figure 7). These results suggest that EGCG and theaflavin can inhibit 3CL-protease without significant cellular cytotoxicity.

## 4. Discussion

The discovery for a COVID-19 treatment is urgent as COVID-19 spreads very rapidly, and infections and deaths are continuously rising all over the globe. SARS-CoV-2 3CL-protease is a promising target for COVID-19 treatments. Recently, a peptidomimetic chemical *α*-ketoamide was reported to inhibit the SARS-CoV-2 3CL-protease and reduce viral replication in cell culture [10]. However, further time-consuming testing must occur to determine the safety and efficacy of these chemicals.

In this study, we aimed to determine whether EGCG and theaflavin, the major active components of green tea and black tea, have inhibitory activity against 3CL-protease. Green tea and black tea are consumed widely and regularly throughout the world, and their health benefits have been universally lauded. We demonstrated that both EGCG and theaflavin inhibit 3CL-protease activity in a dose-dependent manner. We calculated the IC_50_ of EGCG and theaflavin and found 7.58 *μ*g/ml for EGCG and 8.44 *μ*g/ml for EGCG (Figures 3 and 4). Theaflavin inhibits SARS 3CL-protease with an IC_50_ value of 7 *μ*g/ml, and various tea extracts were assayed against SARS coronavirus 3CL-protease, with IC_50_ values in the range of 25∼125 *µ*g/ml [21]. In addition, black tea, an oxidized form of green tea, neutralizes bovine coronavirus infectivity at an EC_50_ of 34.7 *μ*g/ml [24]. The inhibitory activity of EGCG and theaflavin on SARS-CoV-2 3CL-protease is consistent with the inhibitory activity of SARS 3CL-protease observed in a previous study [21]. In addition, the purified single compounds (EGCG and theaflavin) show lower IC_50_ than tea extracts, suggesting that EGCG and theaflavin are the main active ingredients that inhibit 3CL-protease.

One major question is whether effective levels of either EGCG or theaflavin can be reached in the body. When we calculated the IC_50_ using the molar concentration of EGCG or theaflavin, the IC_50_ is equal to 16.5 *μ*M for EGCG and 15.0 *μ*M for theaflavin. However, the maximum blood concentration of EGCG is less than 1 *μ*M and the maximum blood concentration of theaflavin is less than 0.1 *μ*M [32–34]. The effect of EGCG or theaflavin, therefore, on coronavirus replication in the human body may be limited on their own. However, our data also showed that EGCG and theaflavin treatment together has an additive effect (Figure 5). Green tea or black tea may provide various EGCG and theaflavin derivatives, and their additive effect may be effective to limit coronavirus replication. Moreover, EGCG can be oxidized in physiological conditions of body and converted into EAOPs. Here, we showed that EAOPs retain inhibitory activity on the 3CL-protease, suggesting that EGCG can be effective for a long time (Figure 6).

In addition, recent computational studies support that EGCG and theaflavin are inhibitors of SARS-CoV-2 3CL-protease. An *in silico* molecular docking study with SARS-CoV-2 3CL-protease showed that EGCG and theaflavin strongly interact with 3CL-protease, indicating that EGCG and theaflavin potentially inhibit SARS-CoV-2 3CL-protease [35–37].

Here, we showed that EGCG and theaflavin, the active ingredients of green tea and black tea, are effective to inhibit the 3CL-protease *in vitro*. Because green tea and black tea contain a high percent of EGCG and theaflavin, it would be valuable to examine the effect of green tea and black tea on the spread of SARS-CoV-2 *in vivo*. In addition, further clinical trials will be required to reveal the effect of tea consumption on COVID-19 prognosis.

## Data Availability

The original data that support the findings of this study are included in the article.

## Abbreviations

COVID-19: 2019 novel coronavirus disease
SARS-CoV-2: Severe acute respiratory syndrome coronavirus 2
3CL-protease: 3C-like protease
EGCG: (−)-Epigallocatechin-3-gallate.
